# Progression to Type 2 Diabetes in Women with Former Gestational Diabetes: Time Trajectories of Metabolic Parameters

**DOI:** 10.1371/journal.pone.0050419

**Published:** 2012-11-21

**Authors:** Andrea Tura, Angela Grassi, Yvonne Winhofer, Annamaria Guolo, Giovanni Pacini, Andrea Mari, Alexandra Kautzky-Willer

**Affiliations:** 1 Metabolic Unit, Institute of Biomedical Engineering, National Research Council, Padova, Italy; 2 Unit of Gender Medicine, Division of Endocrinology and Metabolism, Clinic of Internal Medicine III, Medical University of Vienna, Vienna, Austria; 3 Department of Economics, University of Verona, Verona, Italy; Scientific Directorate, Bambino Hospital, Italy

## Abstract

Aim of this study was analyzing the time trajectories of the metabolic parameters in European women with former gestational diabetes (fGDM), and determining predictors of type 2 diabetes onset. A group of seventy-six fGDM women were studied at the outpatient department of the University Clinic of Vienna. They were evaluated yearly with a 3 h-oral glucose tolerance test (OGTT) up to 7-years from delivery. At baseline, women also underwent an intravenous glucose tolerance test (IVGTT). Insulin sensitivity and beta-cell function were assessed by both OGTT and IVGTT. Women were divided into progressors (PROG) to diabetes (*n* = 19) and non-progressors (*n* = 57). Time trajectories of glycemia and other parameters were analyzed after synchronization to time of diabetes onset or last OGTT. Then, Cox proportional hazard regression analysis was performed to assess the predictive power of studied variables for diabetes onset. We found that, in PROG, time trajectories of glycemia were flat until diabetes onset, when they showed a marked increase (P<0.0001). Insulin sensitivity showed similar marked decrease (P<0.0001) at diabetes onset, together with a tendency to continuous slow decline in the previous years. At contrast, beta-cell function showed only continuous slow decline. Major predictors of diabetes onset were glycemic levels, BMI, insulin resistance, and condition of impaired glucose tolerance. In conclusion, in fGDM, marked deterioration of insulin sensitivity is associated with diabetes onset. Prevention strategies aimed at opposing to the insulin sensitivity derangement may be particularly beneficial.

## Introduction

History of previous gestational diabetes is known to be a risk condition for later development of type 2 diabetes [1]. High glycemic levels during pregnancy or after delivery, both at fasting and during an oral glucose tolerance test (OGTT) are strong predictors of diabetes development in women with former gestational diabetes (fGDM) [2]–[6]. Relative hyperglycemia typically indicates some degree of insulin resistance and beta-cell dysfunction, which can be observed in fGDM women, even with normal body weight and glucose tolerance [7], [8]. However, the role of these factors is still incompletely understood, also due to the differences in methodologies and subjects selection. Specifically, it has not been yet clarified what is the crucial factor, if any, leading to the onset of type 2 diabetes.

In this longitudinal study, we have determined the time trajectories of glycemia, beta-cell function and insulin sensitivity parameters in a group of fGDM women that were nondiabetic at the time of first analysis. We have also evaluated the baseline predictors of diabetes onset.

## Materials and Methods

### Subjects

The study was approved by the local ethics committee (Ethics Committee of the Medical University of Vienna) and was performed in accordance with the Declaration of Helsinki. This was an observational study of descriptive character. The main aims of the study were investigating the changes over time of different metabolic parameters (namely, insulin secretion, beta-cell function and insulin sensitivity), and observing possible development of type 2 diabetes, in women with a recent history of gestational diabetes.

Women were recruited from our outpatient department (Department of Internal Medicine III). All subjects gave written informed consent for participation in the study. A group of 76 Central European fGDM women, diagnosed according to the criteria of the 4^th^ Workshop Conference of Gestational Diabetes [9], were studied. The study started early postpartum (4–6 months after delivery), then all women were reevaluated yearly (with a tolerance of ±2 months) for up to seven years. Subjects analyzed in this study were part of a larger group of fGDM women (*n* = 120); in this investigation we selected those women matching the following criteria: *a)* nondiabetic condition at the time of first analysis (early postpartum), according to the American Diabetes Association 2003 criteria [10] (7 subjects excluded); *b)* follow-up of at least three years, except for subjects developing type 2 diabetes in a shorter time interval (16 subjects excluded); *c)* no further pregnancies during the follow-up period of each subject (18 subjects excluded); *d)* measure of glucose, insulin and C-peptide during an OGTT at each evaluation that the subject underwent (3 subjects excluded). As regards the exclusion criterion *b)*, the three years threshold was used to limit the possibility that some subjects were classified as non-progressors to type 2 diabetes only because they were not observed for a sufficient time period. However, the analysis of the diabetes predictors, based on Cox proportional hazard regression (see the section on statistical analyses), was repeated also including the subjects with short follow-up and with further pregnancies (points *b)* and *c)*, respectively).

All women were islet cell antibodies negative. During the study period, no women took any drug known to affect glucose metabolism. Also, at recruitment time women agreed not to use oral contraceptives for the whole duration of the study. Some of the studied subjects were included in previous analyses [7]–[8], [11].

### Tests

At all visits during the 7-year study, women underwent a standard 75 g OGTT in the morning after an overnight fast. Venous blood samples were collected at fasting and at 10, 20, 30, 60, 90, 120, 150 and 180 min afterwards.

At the baseline visit, all the subjects underwent also an insulin-modified intravenous glucose tolerance test (IVGTT). Glucose was injected at time 0–0.5 min (300 mg/kg) and insulin (0.03 IU/kg, Humulin R; Eli Lilly, Indianapolis, IN) was infused intravenously at time 20 for 5 min. Venous blood samples for determination of plasma concentration of glucose, insulin and C-peptide were collected at fasting and frequently for 180 minutes after glucose injection. In particular, in the first ten minutes after injection samples were collected at 3, 4, 5, 6, 8, 10 min.

Insulin and C-peptide were determined in duplicate by commercially available radioimmunoassay kits with an interassay coefficient of variation <5%.

At baseline, family history of diabetes was also recorded; systolic and diastolic blood pressure, triglycerides and cholesterol were determined.

### Beta-cell function and insulin sensitivity

We assessed beta-cell function by mathematical modeling [12]. In the model, insulin secretion is described as the sum of two components, S_g_(t) and S_d_(t). The former represents the dependence of insulin secretion on absolute glucose concentration (G), and is characterized by a curvilinear dose-response function, f(G). The dose-response is modulated by a time-dependent potentiation factor, P(t); thus, S_g_(t) = P(t)f(G). The second insulin secretion component, S_d_(t), represents a dynamic dependence of secretion on the rate of change of glucose, and is termed derivative component. S_d_(t) is proportional to the glucose time derivative for positive derivative, whereas it is zero otherwise. The most significant component of the insulin secretion is that originating from the beta-cell dose-response: it describes the rise and fall of insulin secretion that parallels the rise and fall of glucose concentration. The derivative component accounts for an initial, fast rise in insulin secretion and is a marker of first phase secretion. The potentiation factor explains the sustained insulin secretion levels that are typically seen at the end of an OGTT in healthy subjects when glucose has already returned to basal. Thus, these three components of the model (dose-response, derivative component and potentiation) represent clearly distinct features of the OGTT secretory response. The main beta-cell function parameters derived from the model are: *a)* beta-cell glucose sensitivity: the mean value of the dose-response slope; *b)* rate sensitivity: the proportionality constant of the derivative component; *c)* ratio of the potentiation at 180 to that at zero minutes: a compact index of the potentiation factor excursions. Other parameters derived from the model are the basal and total insulin secretion, and the insulin secretion at prescribed glucose level (typically, the average fasting level), obtained from the dose-response. To determine the model parameters, the described model of insulin secretion is coupled with the standardized model of C-peptide kinetics developed by Van Cauter *et al.* [13]. The combination of the two models represents a relationship between glucose and C-peptide concentration over time, i.e., the experimental data. The model parameters are determined from the glucose and C-peptide data using least squares methods [12].

Beta-cell function from the OGTT was also estimated using the insulinogenic index, IGI, despite its known limitations [14]; it was computed as the ratio of the difference between insulin at 30 min and at fasting to the corresponding difference in glucose.

A beta-cell function index from the IVGTT is the acute insulin response (AIR) [15], computed as the suprabasal integral of plasma insulin in the 0–8 minutes interval normalized to the interval length. Similar parameters was computed from plasma C-peptide (ACPR).

Insulin sensitivity was estimated by OGIS model from the OGTT [16] and Minimal Model analysis from the IVGTT [17]. HOMA-R was also computed as the product of fasting glucose and insulin, divided by 22.5.

### Statistical analysis

Since during the study some women developed type 2 diabetes [10], women were divided into two groups: those progressing to diabetes (progressors, PROG) and those remaining nondiabetic (non-progressors, NONPROG). We tested the statistical significance of the difference in parameter mean values between the PROG and NONPROG groups with the Mann Whitney test and the χ^2^ test as appropriate.

Time trajectories of the metabolic parameters in the PROG and NONPROG groups were plotted and analyzed after synchronization to the last OGTT in the NONPROG group, and to the OGTT at which diabetes was diagnosed in the PROG group. Thus, year 0 represents time of last examination for NONPROG, and time of diabetes onset for PROG. In PROG, no further data were considered after the diagnosis of diabetes. For each time trajectory, we tested the possible difference in the mean value of the metabolic parameter between pairs of study years (that is, between year −5 and −4, year −4 and −3, etc.). To this aim, we take advantage of the generalized estimating equation approach (GEE) [18], as a general tool to account for repeated measures. Another analysis was performed, again based on GEE approach, to evaluate the possible change with time, on average, of the metabolic parameters. These specific analyses were performed in the R programming language by exploiting the functionalities of the geepack package [19].

Cox proportional hazard regression was used to test for possible association between the metabolic variables at baseline and time to diabetes development, and it was expressed in terms of hazard ratios (HR, with 95% confidence intervals, CI). Kaplan-Meier plots were used to compare diabetes-free survival curves by means of the log-rank χ^ 2^ statistics.

This Cox proportional hazard regression analysis was initially performed on the original population (*n* = 76). However, a second analysis was also performed including those women, not progressing to diabetes, which were excluded for the short follow-up or for subsequent pregnancy (see Subjects sections, points *b)*, *n* = 16, and *c)*, *n* = 18, respectively). In this second analysis, women with subsequent pregnancy were censored at the time of such new pregnancy.

Data and results are given as mean±standard error (SE). A value of P<0.05 was considered statistically significant. There was no adjustment for multiple statistical testing.

## Results

### Baseline characteristics

The main characteristics and metabolic parameters of the subjects at baseline are reported in Table 1. Within the 7-years observation period, 25% of the women (*n* = 19 of *n* = 76) progressed to type 2 diabetes. Median follow-up in NONPROG was 5 years; in PROG it was 3 years. In fact, 12 of 19 women in the PROG group developed diabetes within 3 years. The incidence rate of diabetes, expressed in person-years, was 5.54%.

**Table 1:** Baseline characteristics (mean±SE) of subjects progressing to type 2 diabetes (PROG) and not progressing (NONPROG).

	PROG	NONPROG	Whole cohort
*Main characteristics*			
*n*	19	57	76
Age (years)	36.0±1.1 *,†	33.3±0.6	34.0±0.5
BMI (kg/m^2^)	31.7±1.5 *,†	25.3±0.5	26.9±0.6
Impaired glucose regulation, IGR (*n*)	13/19 *,†	4/57	17/76
*Plasma concentration levels (OGTT)*			
Fasting glucose (mmol/l)	5.44±0.14 *,†	4.78±0.05	4.92±0.06
2 h glucose (mmol/l)	8.19±0.41 *,†	5.99±0.16	6.54±0.20
Mean glucose (mmol/l)	8.26±0.26 *,†	6.92±0.18	7.27±0.17
Fasting insulin (pmol/l)	78±11 *,†	49±4	56±4
Mean insulin (pmol/l)	353±48 *	287±22	305±21
Fasting C-peptide (pmol/l)	810±79 *,†	544±28	602±32
Mean C-peptide (pmol/l)	2499±157 *	2200±90	2284±82
*Beta-cell function and insulin secretion*			
Glucose sensitivity (OGTT) (pmol min^−1^ m^−2^ mM^−1^)	73±9 *,†	103±5	96±5
Rate sensitivity (OGTT) (pmol m^−2^ mM^−1^)	299±89 *,†	678±65	584±58
Potentiation factor ratio (dimensionless)	1.42±0.07	1.45±0.06	1.43±0.05
Fasting insulin secretion (OGTT) (pmol min^−1^ m^−2^)	107±9 *,†	77±4	83±4
Total insulin secretion (OGTT) (nmol m^−2^)	66±4 *	57±3	58±2
Insulin secretion at 5.5 mmol/l gluc. (OGTT) (pmol min^−1^ m^−2^)	180±20	205±10	199±9
Insulinogenic index, IGI (OGTT) (pmol/mmol)	62±15	105±25	93±20
Acute insulin response, AIR (IVGTT) (pmol/l)	153±37	189±18	181±17
Acute C-peptide response, ACPR (IVGTT) (pmol/l)	544±102	688±48	655±47
*Insulin sensitivity*			
Insulin sensitivity, OGIS (OGTT) (ml min^−1^ m^−2^)	382±12 *,†	456±9	441±9
Insulin sensitivity, S_I_ (IVGTT) (10^−4^ min^−1^ (µU/ml)^−1^)	2.65±0.48 *	4.08±0.36	3.75±0.33
Insulin resistance, HOMA-R (dimensionless)	3.13±0.42 *	2.12±0.18	2.39±0.19
*Other parameters*			
Systolic blood pressure (mmHg)	122±4 *,†	111±2	114±2
Diastolic blood pressure (mmHg)	79±3	77±1	77±1
Triglycerides (mg/dl)	132±12 *	103±14	109±11
Cholesterol (mg/dl)	205±9	212±6	208±4
High density lipoprotein cholesterol, HDL (mg/dl)	47.7±3.7 *,†	63.8±3.2	59.2±2.7
Family history of diabetes (*n*)	14/19 *	28/57	42/76
Insulin during pregnancy (*n*)	17/19 *,†	36/57	53/76

Values of the variables in the whole cohort are also reported.

* Significant difference between PROG and NONPROG

† Significant diabetes predictor in univariate Cox proportional hazard regression analysis

Although both PROG and NONPROG were nondiabetic at baseline, PROG had higher fasting, 2 h and mean glucose levels at the OGTT. Consequently, 13 out of 19 PROG had impaired glucose regulation (impaired fasting glucose and/or impaired glucose tolerance [10]), whereas impaired glucose regulation was present only in 4 of the 57 NONPROG women. PROG also had higher insulin and C-peptide levels. In PROG, beta-cell function and insulin sensitivity were impaired. PROG also exhibited higher BMI, triglycerides levels and systolic blood pressure. Finally, PROG had more frequent family history of diabetes.

### Time trajectories

**Figure 1:**
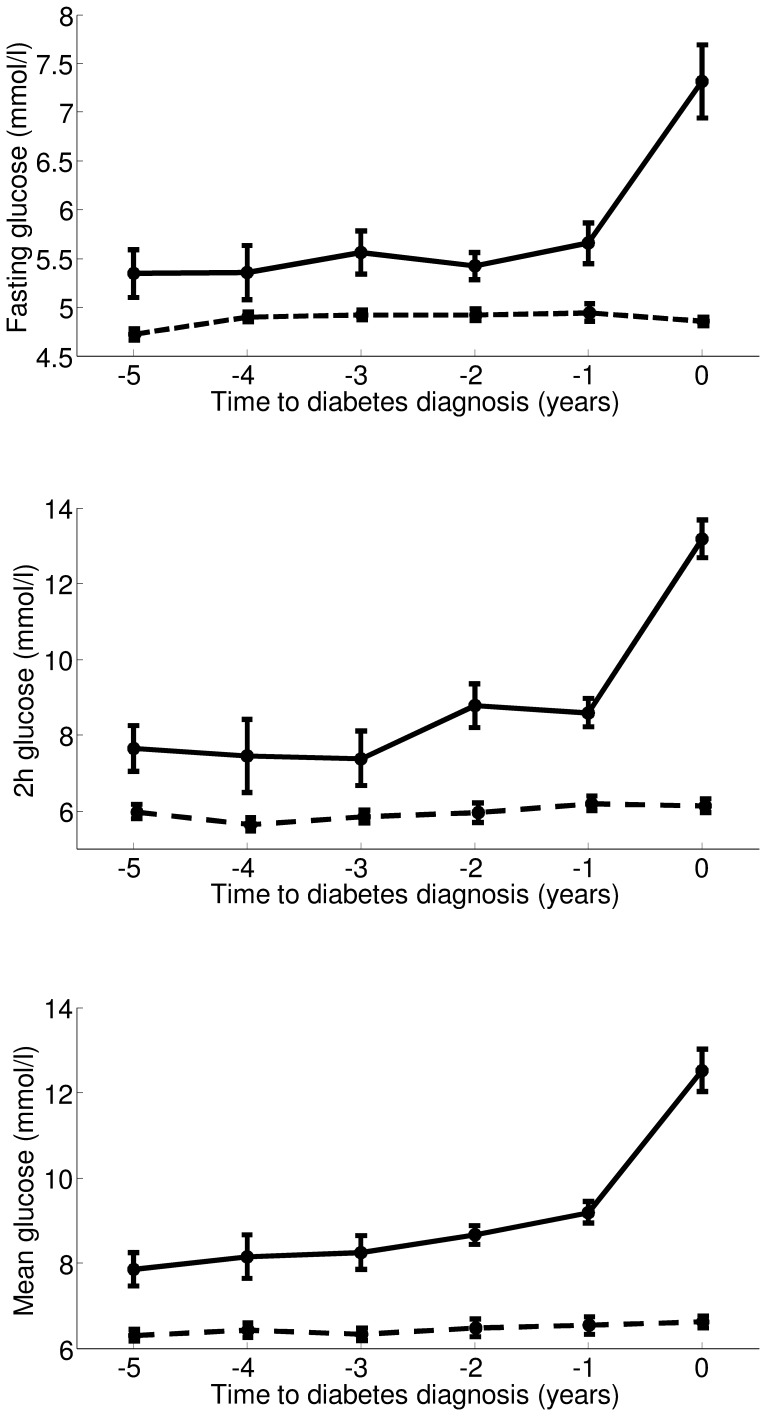
Time trajectories of glycemic levels. Fasting glucose (top), 2 h glucose (center) and mean glucose (bottom) in PROG (solid line) and NONPROG (dashed line). Data (mean±SE) are reported from year −5 to year 0 (time of diabetes onset for PROG, and of last examination for NONPROG). In PROG, the number of subjects at each time sample is: 6, 6, 9, 10, 13, 19 (from year −5 to year 0, respectively); in NONPROG: 45, 51, 48, 38, 25, 57, respectively.

In PROG, fasting glucose remained essentially constant for several years and then showed a sharp increase between year −1 and year 0, i.e., when type 2 diabetes was diagnosed (Figure 1, top). Similar trajectories were observed also for 2 h glucose (Figure 1, center) and mean OGTT glucose (Figure 1, bottom). Fasting, 2 h, and mean glucose values at year 0 were significantly higher than the corresponding values at year −1 (P<0.0001 by GEE analysis for all parameters). Mean glucose also showed an increase between year −2 and year −1 (P<0.02). In NONPROG, glucose values were essentially stable during the observation period (Figure 1).

**Figure 2:**
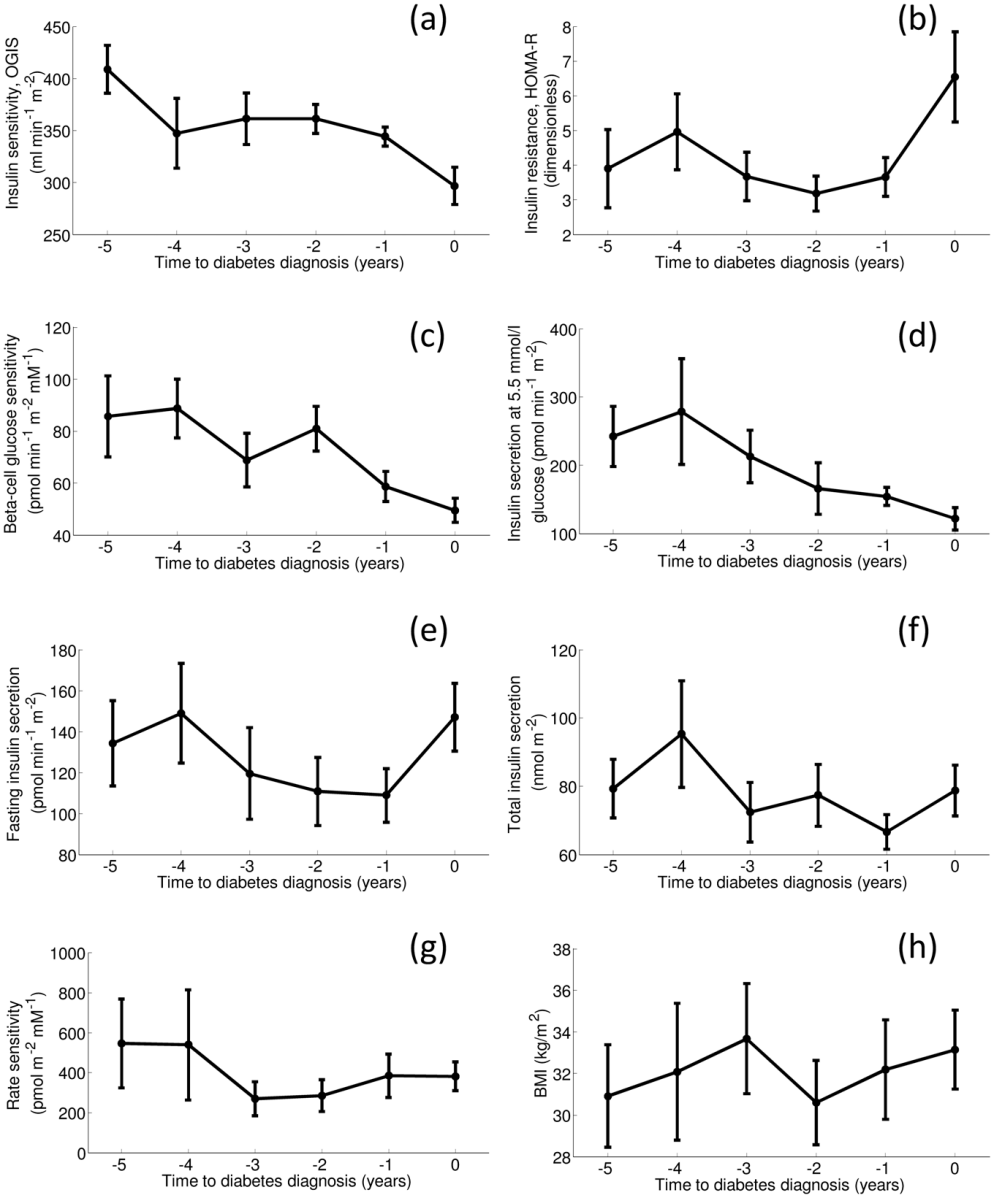
Time trajectories of the main metabolic parameters. OGIS (a), HOMA-R (b), beta-cell glucose sensitivity (c), insulin secretion at 5.5 mmol/l glucose level (d), fasting insulin secretion (e), total insulin secretion (f), rate sensitivity (g), and BMI (h), in PROG. Data (mean±SE) are reported from year −5 to year 0 (time of diabetes onset). The number of subjects at each time sample is: 6, 6, 9, 10, 13, 19 (from year −5 to year 0, respectively).

As regards the main metabolic parameters, insulin sensitivity from the OGTT, OGIS, showed in PROG a marked decrease (P<0.0001) between year −1 and year 0 (Figure 2, a). Similar results were obtained with HOMA-R (P = 0.02) (Figure 2, b), and also with other OGTT-based indices, such as MCR_est_ [20], ISI(comp) [21], S_I(oral)_ [22] (results not shown). At contrast, for both OGIS and HOMA-R significant variations between the previous pairs of years were not observed, not even between year −5 and year −4. GEE analysis of the possible average change with time showed borderline or not significant P values (P≥0.056). Thus, insulin sensitivity is clearly characterized by a marked decrease at the diabetes onset; as regards a possible less marked, but continuous decline in the preceding years, results suggest a tendency to decline, but from our data it cannot be completely elucidated whether such slow decline during years is in fact really significant.

Beta-cell glucose sensitivity and insulin secretion at 5.5 mmol/l glucose (a value close to average fasting glucose in PROG) did not show significant variations between pairs of years, but visual inspection of the time trajectories suggests a tendency to a continuous decrease (Figure 2, c and d). In fact, GEE analysis found a significant decrease over time, on average, for insulin secretion at 5.5 mmol/l (P<0.01), whereas such time change did not reach statistical significance for glucose sensitivity.

Absolute fasting and total insulin secretion showed similar tendency to decrease (Figure 2, e and f), except at year 0 when the glucose levels rose substantially. GEE analysis of year-to-year variations found for both parameters a significant increase between year −1 and 0 (P<0.01), but also provided some uncertain results for the previous years (for total secretion, a decrease between year −4 and −3, followed by an increase between −3 and −2, P<0.03). Rate sensitivity remained essentially constant (Figure 2, g). BMI did not show a systematic trend to increase (Figure 2, h), though GEE analysis showed a slight increase (P = 0.036) between year −2 and year −1.

BMI, beta-cell function and insulin sensitivity parameters in NONPROG did not show significant changes during the whole observation period.

### Cox proportional hazard regression analysis and Kaplan-Meier plots

In the Cox proportional hazard regression analysis, we considered all the variables reported in Table 1. In univariate analysis, many of these variables were significantly associated with diabetes development (see Table 1). Our findings showed that increased glycemic levels or BMI, and decreased insulin sensitivity, were particularly relevant for the risk of developing diabetes. In fact, among the continuous parameters, the strongest predictors of diabetes development were glycemic levels, BMI, insulin sensitivity expressed by OGIS (P<0.0001 for all variables). The hazard ratio per 1 standard deviation for such variables was HR_FAST_GLU_ = 2.99 (95% CI: 1.96–4.57), HR_2H_GLU_ = 3.40 (2.10–5.52), HR_MEAN_GLU_ = 2.49 (1.47–4.22), HR_BMI_ = 2.19 (1.54–3.13), HR_OGIS_ = 0.28 (0.15–0.53). Other strong predictors of diabetes were beta-cell glucose sensitivity (HR_GLU_SENS_ = 0.41 (0.23–0.75)), HDL (HR_HDL_ = 0.32 (0.15–0.71)), fasting C-peptide (HR_FAST_CPEP_ = 1.63 (1.18–2.25)), P<0.01 for all. Of note, high fasting C-peptide levels were associated with an increased risk of diabetes, and this was confirmed by similar findings on fasting insulin levels and fasting insulin secretion, though with somehow higher P values (P = 0.014 for both). At contrast, mean insulin and C-peptide values during the OGTT, and total insulin secretion, were not significant predictors of diabetes risk (P>0.15). Among categorical variables, the condition of impaired glucose regulation was strongly associated with diabetes development, thus confirming the findings on glycemic levels (HR_IGR_ = 14.54 (5.13–41.21), P<0.0001). Also the intake of insulin medication during pregnancy was a relatively strong predictor of diabetes (HR_INS_PREGN_ = 11.01 (1.47–82.68), P<0.02).

**Figure 3:**
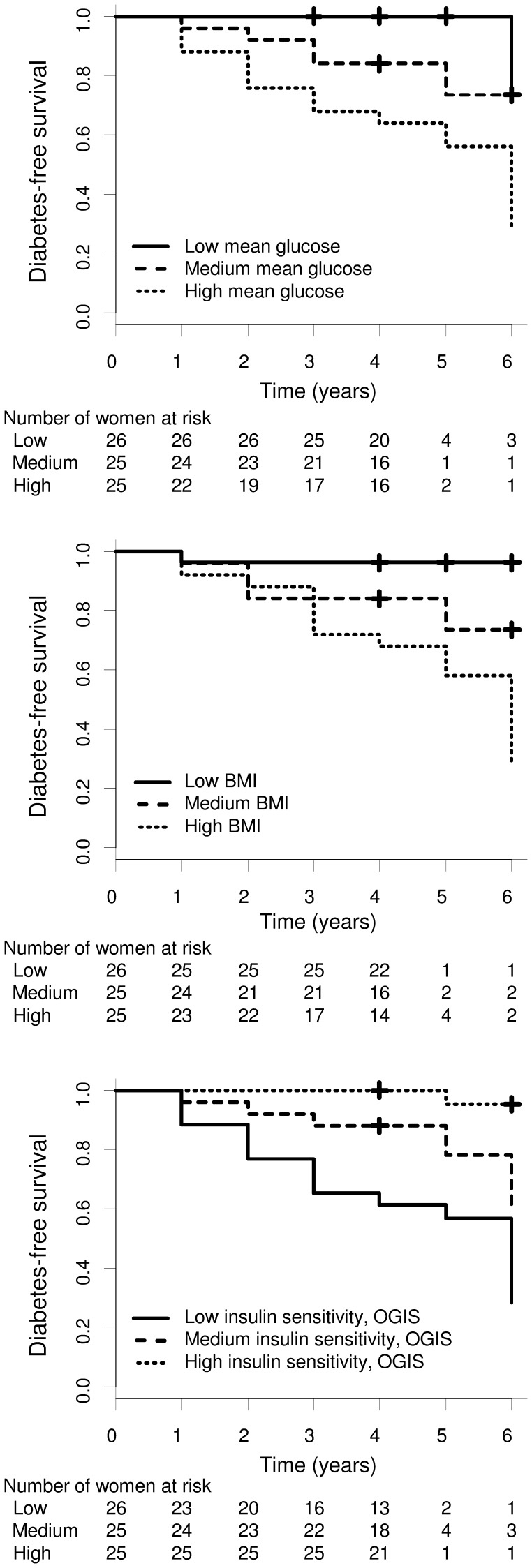
Kaplan-Meier plots of diabetes-free survival. Plots refer to mean glucose (top), BMI (medium), insulin sensitivity (bottom). For each variable, subjects have been stratified for tertiles (low, medium, high). Numbers of subjects at risk in each stratum at each year have been reported. Log-rank χ^2^ statistics was 13.3 (P = 0.001) for mean glucose, 10.7 (P = 0.005) for BMI, 12.6 (P = 0.002) for insulin sensitivity.

Kaplan-Meier plots of diabetes-free survival for some of the strongest diabetes predictors, i.e., mean glucose, BMI and OGIS, are reported in Figure 3. The subjects have been stratified for tertiles (low, medium, high) of each variable values. The plots clearly confirm the role of each of the three variables as predictor of diabetes onset. In fact, 48% (*n* = 12 of 25) of the subjects in the high tertile of mean glucose or BMI, or in the low tertile of OGIS, developed type 2 diabetes.

Cox proportional hazard regression analysis was also repeated including the subjects with short follow-up period, and with subsequent pregnancy (censored at the time of the new pregnancy). This new analysis completely confirmed the previous results. The strongest predictors of diabetes development (P<0.0001 for all) remained in fact the same of the previous analysis: glycemic levels (HR_FAST_GLU_ = 3.01 (95% CI: 1.96–4.63), HR_2H_GLU_ = 3.27 (2.06–5.19), HR_MEAN_GLU_ = 2.47 (1.49–4.09)), BMI (HR_BMI_ = 2.17 (1.52–3.09)), OGIS (HR_OGIS_ = 0.27 (0.14–0.51)). Other strong predictors of diabetes (P<0.01 for all) were again beta-cell glucose sensitivity (HR_GLU_SENS_ = 0.37 (0.19–0.71)), HDL (HR_HDL_ = 0.37 (0.18–0.75)), fasting C-peptide (HR_FAST_CPEP_ = 1.69 (1.18–2.42)). Among the categorical variables, both impaired glucose regulation and insulin intake during pregnancy were confirmed being associated with diabetes development (HR_IGR_ = 14.64 (5.16–41.48), P<0.0001, HR_INS_PREGN_ = 11.07 (1.47–83.20), P<0.02).

## Discussion

Several studies have investigated the factors predicting the development of type 2 diabetes in women with a history of gestational diabetes. However, in many studies only glycemic levels were analyzed, possibly with the addition of few basic clinical variables and anthropometric data [11], [23]–[27]. Other studies performed more detailed analyses, also including indices of insulin sensitivity or beta-cell function, but the follow-up period was short, being limited to 1–2 years [28], [29].

Some recent studies partially overcame the main limitations of the previous analyses [30]–[32]. In the study by Seghieri *et al.* [30], both indices of insulin sensitivity and beta-cell function were studied. However, surprisingly insulin sensitivity was not found a significant predictor of diabetes development. Similarly, in the study by Ekelund *et al.* [31], the findings about insulin sensitivity were inconclusive. The study by Xiang, Kjos *et al.* [32] reported a detailed analysis of several possible predictors of diabetes development, including indices of insulin sensitivity and beta-cell function from three different glucose tolerance tests, and extending the follow-up period to twelve years. Indices of both insulin sensitivity and beta-cell function were found to be predictors, together with the glycemic levels, of diabetes development [32]. Thus, our findings are in agreement with those of the study [32]. However, in the study [32] no OGTT-based indices of insulin sensitivity or beta-cell function were found to be predictors of diabetes development. In the clinical routine, cumbersome tests such as the IVGTT or the glucose clamp may not be feasible, and this would prevent the application of the findings of study [32] in terms of identification of the subjects at higher risk for later diabetes.

Among the substantial number of previous studies in women with a history of gestational diabetes, to our knowledge none reported detailed analysis of time trajectories of metabolic parameters in fGDM women that actually progressed to type 2 diabetes. This is the main novelty of our study. The only previous study that reported some information on time trajectories in fGDM women was the study by Xiang, Kawakubo *et al.* [33], but the temporal analysis was limited to few metabolic parameters. Besides, in the study [33] the subjects were not stratified between progressors and non-progressors, thus a direct comparison with our results is not possible. Finally and most importantly, our population included selected Central European women, while both studies by Xiang *et al.* [32]-[33] described Hispanic women, which may have metabolic differences compared to our women [34]; furthermore, disparities in the diabetes risk for different populations of fGDM women have been outlined by another recent study by Xiang *et al.* [35].

In our study, we have shown for the first time that the onset of hyperglycemia in fGDM is rapid, as it has been observed in other populations progressing to type 1 or type 2 diabetes [36]–[39]. However, the mechanisms behind the development of hyperglycemia in fGDM appear to be specific, and related to exacerbation of insulin resistance on a background of a considerable impairment in beta-cell function since baseline (i.e., immediately after partum). It should be noted that both defects in insulin sensitivity and beta-cell function were clearly present at baseline in PROG. In fact, beta-cell glucose sensitivity and rate sensitivity (see Table 1) were markedly lower in PROG than in NONPROG, and the difference remained significant after adjusting for age, BMI or both (Fisher's PLSD *post hoc* Analysis of Covariance on logarithmically transformed values: P<0.003 for glucose sensitivity; P<0.01 for rate sensitivity). This is consistent with the findings of a previous study where beta-cell function defect was observed already 4–6 months after delivery in fGDM women that had both normal glucose tolerance and body weight [7]. In addition, analysis of the time trajectories showed that, in PROG, some of the beta-cell function parameters exhibited a tendency to further decline during the observation period. Thus, we conclude that in PROG, beta-cell function showed both baseline impairment and some degree of further deterioration thereafter, and this is in agreement with the findings of study by Xiang, Kjos *et al.* [32]. It should be noted that beta-cell glucose sensitivity is not related to insulin sensitivity [40], thus the pattern represented in Figure 2, c, correctly estimates the degree of beta-cell function deterioration. Insulin secretion at 5.5 mmol/l glucose (Figure 2, d) may require some adjustment for insulin sensitivity [40], but this cannot be easily taken into account, since precise relationship between this parameter and the OGTT insulin sensitivity indices has not been determined. Adjustment for insulin sensitivity may yield a more marked decrease of insulin secretion at 5.5 mmol/l glucose.

Thus, the defects in beta-cell function appear evident in the PROG group. The defects in insulin sensitivity were also evident, since both OGIS and HOMA-R were markedly lower in PROG at baseline, and similarly to beta-cell function parameters they also exhibited a tendency to further decline during years. However, based on our analysis, these defects seem insufficient to explain the onset of diabetes, which occurs only when a rapid, marked deterioration in insulin sensitivity occurs, in correspondence with the rise of glucose levels. To our knowledge, this was not reported in any of the previous studies on fGDM.

As regards the progression towards type 2 diabetes of populations different than fGDM, a major study is that of Tabák *et al.* [38]. In that study, diabetes onset is due to a marked decline in beta-cell function, occurring in a relatively short time, accompanied by hyperglycemia, on a background of progressive decline in insulin sensitivity. It should be noted, however, that in the study [38] an empirical surrogate of beta-cell function was employed, which may not yield the same evaluation of beta-cell function as does our OGTT-based approach. Therefore, although beta-cell dysfunction is a key factor for diabetes development also in our fGDM population, insulin resistance is likely to be the triggering phenomenon.

This observation on the crucial role of insulin sensitivity has potentially important therapeutic implications. In fact, our findings suggest that prevention strategies aimed at opposing to the insulin sensitivity derangement may be particularly beneficial. This may be feasible through lifestyle intervention only [41], whereas preserving beta-cell can be difficult without pharmacological intervention.

Some limitations of our study should be considered. The size of the analyzed population and consequently the number of progressors is limited, due mainly to the strict selection criteria of the analyzed subjects. However, a relatively small population size was also common to other relevant longitudinal studies on fGDM, including studies by Xiang *et al.* [32]–[33]. Another limitation of our study was that it was not possible to confirm our main findings, especially on insulin sensitivity, through tests different than the OGTT. Nonetheless, all the insulin sensitivity indices that we have evaluated are concordant in indicating the pattern observed in Figure 2. It should also be acknowledged that, although our study shows a clear association between insulin sensitivity deterioration and diabetes onset, whether this is a causal relationship cannot be concluded with certainty. In fact, the causes of the deterioration in insulin sensitivity remain unclear. BMI may be one factor; however, a significant BMI change was observed only between year −2 and year −1, and not when the marked deterioration of insulin sensitivity was observed (year −1 to 0). An aspect recently emerging as possible factor affecting insulin sensitivity is breast feeding. Unfortunately, this information was not recorded precisely in our study. We know that all women breast fed at least partially, except for two of them (one in PROG, one in NONPROG), but further information was not collected. On the other hand, at the time of recruitment in the study, the women were not breastfeeding any more. In any case, the effect of breast feeding is still controversial, as recent studies reported either favorable effects on glucose metabolism [42] or no significant effects [43].

In our study, subjects received the diagnosis of gestational diabetes according to criteria that were recommended when the study started [9]. Recently, different criteria have been proposed by the American Diabetes Association [44] and the International Association of Diabetes and Pregnancy Study Groups [45]. However, these new criteria [44]–[45] do not affect the present analysis, since all the subjects analyzed, diagnosed as having gestational diabetes with the old criteria [9], would receive the same diagnosis with the new criteria [44]–[45].

In conclusion, we have studied a selected group of Central European women with former gestational diabetes for a period up to seven years. During the follow-up period, 25% of the women developed type 2 diabetes. The main finding based on the analysis of the time trajectories of the metabolic parameters was that, on a background of impaired and slowly declining beta-cell function and insulin sensitivity, a further marked deterioration of insulin sensitivity is likely to be the crucial factor for the onset of diabetes.

## References

[1] Kim C, Newton KM, Knopp RH (2002) Gestational diabetes and the incidence of Type 2 diabetes: a systematic review. Diabetes Care 25: 1862–1868. 12351492 10.2337/diacare.25.10.1862

[2] Kjos SL, Buchanan TA, Greenspoon JS, Montoro M, Bernstein GS, et al (1990) Gestational diabetes mellitus: the prevalence of glucose intolerance and diabetes mellitus in the first two months postpartum. Am J Obstet Gynecol 163: 93–98. 2375376 10.1016/s0002-9378(11)90676-0

[3] Lam KS, Li DF, Lauder IJ, Lee CP, Kung AW, et al (1991) Prediction of persistent carbohydrate intolerance in patients with gestational diabetes. Diabetes Res Clin Pract 12: 181–186. 1889347 10.1016/0168-8227(91)90075-o

[4] Catalano PM, Vargo KM, Bernstein IM, Amini SB (1991) Incidence and risk factors associated with abnormal postpartum glucose tolerance in women with gestational diabetes. Am J Obstet Gynecol 165: 914–919. 1951553 10.1016/0002-9378(91)90438-w

[5] Kjos SL, Peters RK, Xiang A, Henry OA, Montoso M, et al (1995) Predicting future diabetes in Latino women with gestational diabetes. Utility of early postpartum glucose tolerance testing. Diabetes 44: 586–591. 7729620 10.2337/diab.44.5.586

[6] Steinhart JR, Sugarman JR, Connell FA (1997) Gestational diabetes is a herald of NIDDM in Navajo women. High rate of abnormal glucose tolerance after GDM. Diabetes Care 20: 943–947. 9167104 10.2337/diacare.20.6.943

[7] Tura A, Mari A, Winzer C, Kautzky-Willer A, Pacini G (2006) Impaired beta-cell function in lean normotolerant former gestational diabetic women. Eur J Clin Invest 36: 22–28. 16403006 10.1111/j.1365-2362.2006.01587.x

[8] Tura A, Mari A, Prikoszovich T, Pacini G, Kautzky-Willer A (2008) Value of the intravenous and oral glucose tolerance tests for detecting subtle impairments in insulin sensitivity and beta-cell function in former gestational diabetes. Clin Endocrinol Oxf 69: 237–243. 18194489 10.1111/j.1365-2265.2008.03178.x

[9] Metzger BE, Coustan DR (1998) Summary and recommendations of the Fourth International Workshop-Conference on Gestational Diabetes Mellitus. The Organizing Committee. Diabetes Care 21: B161–B167. 9704245

[10] The Expert Committee on the Diagnosis and Classification of Diabetes Mellitus (2003) Follow-up Report on the Diagnosis of Diabetes Mellitus. Diabetes Care 26: 3160–3167. 14578255 10.2337/diacare.26.11.3160

[11] Göbl CS, Bozkurt L, Prikoszovich T, Winzer C, Pacini G, et al (2011) Early possible risk factors for overt diabetes after gestational diabetes mellitus. Obstet Gynecol 118: 71–78. 21691165 10.1097/AOG.0b013e318220e18f

[12] Mari A, Tura A, Toschi E, Gastaldelli A, Camastra S, et al (2001) Assessing insulin secretion by modeling multiple meal tests: role of potentiation. Diabetes 51: S221–S226. 10.2337/diabetes.51.2007.s221 11815483

[13] Van Cauter E, Mestrez F, Sturis J, Polonsky KS (1992) Estimation of insulin secretion rates from C-peptide levels. Comparison of individual and standard kinetic parameters for C-peptide clearance. Diabetes 41: 368–377. 1551497 10.2337/diab.41.3.368

[14] Tura A, Kautzky-Willer A, Pacini G (2006) Insulinogenic indices from insulin and C-peptide: comparison of beta-cell function from OGTT and IVGTT. Diabetes Res Clin Pract 72: 298–301. 16325298 10.1016/j.diabres.2005.10.005

[15] Johnston C, Raghu P, McCulloch DK, Beard JC, Ward WK, et al (1987) Beta-cell function and insulin sensitivity in nondiabetic HLA-identical siblings of insulin-dependent diabetics. Diabetes 36: 829–837. 3556281 10.2337/diab.36.7.829

[16] Mari A, Pacini G, Murphy E, Ludvik B, Nolan JJ (2001) A model-based method for assessing insulin sensitivity from the oral glucose tolerance test. Diabetes Care 24: 539–548. 11289482 10.2337/diacare.24.3.539

[17] Pacini G, Tonolo G, Sambataro M, Maioli M, Ciccarese M, et al (1998) Insulin sensitivity and glucose effectiveness: minimal model analysis of regular and insulin-modified FSIGT. Am J Physiol Endocrinol Metab 274: E592–E599. 10.1152/ajpendo.1998.274.4.E592 9575818

[18] Liang KY, Zeger S (1986) Longitudinal data analysis using generalized linear models. Biometrika 73: 13–22.

[19] Højsgaard S, Halekoh U, Yan J (2006) The R Package geepack for Generalized Estimating Equations. J Stat Softw 15: 1–11.

[20] Stumvoll M, Mitrakou A, Pimenta W, Jenssen T, Yki-Järvinen H, et al (2000) Use of the oral glucose tolerance test to assess insulin release and insulin sensitivity. Diabetes Care 23: 295–301. 10868854 10.2337/diacare.23.3.295

[21] Matsuda M, DeFronzo RA (1999) Insulin sensitivity indices obtained from oral glucose tolerance testing: comparison with the euglycemic insulin clamp. Diabetes Care 22: 1462–1470. 10480510 10.2337/diacare.22.9.1462

[22] Caumo A, Bergman RN, Cobelli C (2000) Insulin sensitivity from meal tolerance tests in normal subjects: a minimal model index. J Clin Endocrinol Metab 85: 4396–4402. 11095485 10.1210/jcem.85.11.6982

[23] Damm P, Kühl C, Bertelsen A, Mølsted-Pedersen L (1992) Predictive factors for the development of diabetes in women with previous gestational diabetes mellitus. Am J Obstet Gynecol 167: 607–616. 1530012 10.1016/s0002-9378(11)91559-2

[24] Coustan DR, Carpenter MW, O'Sullivan PS, Carr SR (1993) Gestational diabetes: predictors of subsequent disordered glucose metabolism. Am J Obstet Gynecol 168: 1139–1144. 8475959 10.1016/0002-9378(93)90358-p

[25] Kjos SL, Peters RK, Xiang A, Henry OA, Montoro M, et al (1995) Predicting future diabetes in Latino women with gestational diabetes. Utility of early postpartum glucose tolerance testing. Diabetes 44: 586–591. 7729620 10.2337/diab.44.5.586

[26] Metzger BE, Cho NH, Roston SM, Radvany R (1993) Prepregnancy weight and antepartum insulin secretion predict glucose tolerance five years after gestational diabetes mellitus. Diabetes Care 16: 1598–1605. 8299456 10.2337/diacare.16.12.1598

[27] Dalfrà MG, Lapolla A, Masin M, Giglia G, Dalla Barba B, et al (2001) Antepartum and early postpartum predictors of type 2 diabetes development in women with gestational diabetes mellitus. Diabetes Metab 27: 675–680. 11852376

[28] Buchanan TA, Xiang AH, Kjos SL, Trigo E, Lee WP, et al (1999) Antepartum predictors of the development of type 2 diabetes in Latino women 11–26 months after pregnancies complicated by gestational diabetes. Diabetes 48: 2430–2436. 10580433 10.2337/diabetes.48.12.2430

[29] Retnakaran R, Qi Y, Connelly PW, Sermer M, Hanley AJ, et al (2010) Risk of early progression to prediabetes or diabetes in women with recent gestational dysglycaemia but normal glucose tolerance at 3-month postpartum. Clin Endocrinol Oxf 73: 476–483. 20550531 10.1111/j.1365-2265.2010.03834.x

[30] Seghieri G, Tesi F, De Bellis A, Anichini R, Fabbri G, et al (2010) Long term predictors of post-partum glucose metabolism in women with gestational diabetes mellitus. Exp Clin Endocrinol Diabetes 118: 485–489. 20361392 10.1055/s-0030-1249634

[31] Ekelund M, Shaat N, Almgren P, Groop L, Berntorp K (2010) Prediction of postpartum diabetes in women with gestational diabetes mellitus. Diabetologia 53: 452–457. 19957074 10.1007/s00125-009-1621-3

[32] Xiang AH, Kjos SL, Takayanagi M, Trigo E, Buchanan TA (2010) Detailed physiological characterization of the development of type 2 diabetes in Hispanic women with prior gestational diabetes mellitus. Diabetes 59: 2625–2630. 20682697 10.2337/db10-0521 PMC3279539

[33] Xiang AH, Kawakubo M, Trigo E, Kjos SL, Buchanan TA (2010) Declining beta-cell compensation for insulin resistance in Hispanic women with recent gestational diabetes mellitus: association with changes in weight, adiponectin, and C-reactive protein. Diabetes Care 33: 396–401. 19933993 10.2337/dc09-1493 PMC2809290

[34] Lanting LC, Joung IM, Mackenbach JP, Lamberts SW, Bootsma AH (2005) Ethnic differences in mortality, end-stage complications, and quality of care among diabetic patients: a review. Diabetes Care 28: 2280–2288. 16123507 10.2337/diacare.28.9.2280

[35] Xiang AH, Li BH, Black MH, Sacks DA, Buchanan TA, et al (2011) Racial and ethnic disparities in diabetes risk after gestational diabetes mellitus. Diabetologia 54: 3016–3021. 22016046 10.1007/s00125-011-2330-2

[36] Ferrannini E, Nannipieri M, Williams K, Gonzales C, Haffner SM, et al (2004) Mode of onset of type 2 diabetes from normal or impaired glucose tolerance. Diabetes 53: 160–165. 14693710 10.2337/diabetes.53.1.160

[37] Mason CC, Hanson RL, Knowler WC (2007) Progression to type 2 diabetes characterized by moderate then rapid glucose increases. Diabetes 56: 2054–2061. 17473220 10.2337/db07-0053

[38] Tabák AG, Jokela M, Akbaraly TN, Brunner EJ, Kivimäki M, et al (2009) Trajectories of glycaemia, insulin sensitivity, and insulin secretion before diagnosis of type 2 diabetes: an analysis from the Whitehall II study. Lancet 373: 2215–2221. 19515410 10.1016/S0140-6736(09)60619-X PMC2726723

[39] Ferrannini E, Mari A, Nofrate V, Sosenko JM, Skyler JS, et al (2010) Progression to diabetes in relatives of type 1 diabetic patients: mechanisms and mode of onset. Diabetes 59: 679–685. 20028949 10.2337/db09-1378 PMC2828663

[40] Mari A, Tura A, Natali A, Laville M, Laakso M, et al (2010) Impaired beta cell glucose sensitivity rather than inadequate compensation for insulin resistance is the dominant defect in glucose intolerance. Diabetologia 53: 749–756. 20225397 10.1007/s00125-009-1647-6

[41] Ratner RE, Christophi CA, Metzger BE, Dabelea D, Bennett PH, et al (2008) Diabetes Prevention Program Research Group. Prevention of diabetes in women with a history of gestational diabetes: effects of metformin and lifestyle interventions. J Clin Endocrinol Metab 93: 4774–4779. 18826999 10.1210/jc.2008-0772 PMC2626441

[42] Gunderson EP, Hedderson MM, Chiang V, Crites Y, Walton D, et al (2012) Lactation intensity and postpartum maternal glucose tolerance and insulin resistance in women with recent GDM: the SWIFT cohort. Diabetes Care 35: 50–56. 22011407 10.2337/dc11-1409 PMC3241296

[43] Kim SH, Kim MY, Yang JH, Park SY, Yim CH, et al (2011) Nutritional risk factors of early development of postpartum prediabetes and diabetes in women with gestational diabetes mellitus. Nutrition 27: 782–788. 21106349 10.1016/j.nut.2010.08.019

[44] American Diabetes Association (2012) Diagnosis and classification of diabetes mellitus. Diabetes Care 35: S64–S71. 22187472 10.2337/dc12-s064 PMC3632174

[45] International Association of Diabetes and Pregnancy Study Groups Consensus Panel (2010) Metzger BE, Gabbe SG, Persson B, Buchanan TA, et al (2010) International association of diabetes and pregnancy study groups recommendations on the diagnosis and classification of hyperglycemia in pregnancy. Diabetes Care 33: 676–682. 20190296 10.2337/dc09-1848 PMC2827530

